# Focal Adhesion Kinase and Colony Stimulating Factors: Intestinal Homeostasis and Innate Immunity Crosstalk

**DOI:** 10.3390/cells13141178

**Published:** 2024-07-11

**Authors:** Nicholas D. Brown, Emilie E. Vomhof-DeKrey

**Affiliations:** 1 Department of Pathology, School of Medicine and the Health Sciences, University of North Dakota, Grand Forks, ND 58203, USA; nicholas.d.brown@und.edu; 2 Department of Surgery, School of Medicine and the Health Sciences, University of North Dakota, Grand Forks, ND 58203, USA; 3 Department of Biomedical Sciences, School of Medicine and the Health Sciences, University of North Dakota, Grand Forks, ND 58203, USA

**Keywords:** macrophages, epithelial migration, GM-CSF, M-CSF, wound healing, intestinal injury, cell mobility, chemotaxis

## Abstract

Thousands struggle with acute and chronic intestinal injury due to various causes. Epithelial intestinal healing is dependent on phenotypic transitions to a mobile phenotype. Focal adhesion kinase (FAK) is a ubiquitous protein that is essential for cell mobility. This phenotype change is mediated by FAK activation and proves to be a promising target for pharmaceutical intervention. While FAK is crucial for intestinal healing, new evidence connects FAK with innate immunity and the importance it plays in macrophage/monocyte chemotaxis, as well as other intracellular signaling cascades. These cascades play a part in macrophage/monocyte polarization, maturation, and inflammation that is associated with intestinal injury. Colony stimulating factors (CSFs) such as macrophage colony stimulating factor (M-CSF/CSF-1) and granulocyte macrophage colony stimulating factor (GM-CSF/CSF-2) play a critical role in maintaining homeostasis within intestinal mucosa by crosstalk capabilities between macrophages and epithelial cells. The communication between these cells is imperative in orchestrating healing upon injury. Diving deeper into these connections may allow us a greater insight into the role that our immune system plays in healing, as well as a better comprehension of inflammatory diseases of the gut.

## 1. Intestinal Homeostasis, Injury, and Repair

**Figure 1:**
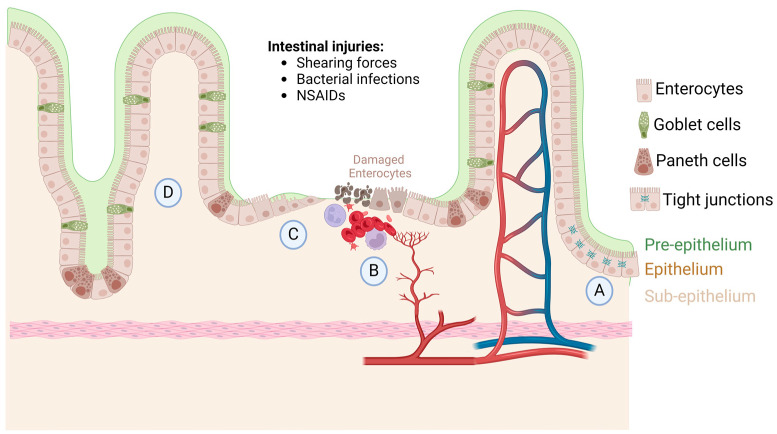
The gastrointestinal epithelium homeostasis, injury, and repair. (**A**) There are three protective layers: pre-epithelium, epithelium, and sub-epithelium. These layers function in epithelial defense and permeability. The tight junctions between mucosal cells act as barriers to bacteria and noxious substance infiltration. (**B**) Platelets are recruited to the injury site where clotting will occur. (**C**) During epithelial restitution, cytokines and immune cells are recruited to the wound to aid in healing. (**D**) IL-22 has been shown to increase the proliferation of Paneth cells and the mucous production of goblet cells, which helps to improve the intestinal membrane defense. Created with BioRender.com (accessed on 29 April 2024).

Intestinal mucosa homeostasis is imperative for proper nutrient absorption. Absorption is carried out by enterocytes and colonocytes in the surface epithelium [1]. The gastrointestinal epithelium has three protective levels: pre-epithelium, epithelium, and sub-epithelium (Figure 1A). The pre-epithelium level contains mucous secretion, bicarbonate, phospholipids, prostaglandins, and trefoil peptides [1,2]. Phospholipids and mucous are secreted onto the epithelium to provide a buffer between the intestinal lumen and the epithelium. The hydrophobicity of these compounds allows for a protective effect [1,2]. Bicarbonate protects the mucosa by neutralizing acid and deactivating digestive enzymes from the gut lumen that are present from stomach chyme. This acid base reaction at the surface of the mucosa provides a near neutral pH at the lumen interface [1,3,4]. Prostaglandins play an important role in protection by inhibiting acid secretion as well as increasing mucous and bicarbonate secretion. They also play an important role in epithelial defense by reducing epithelial permeability [1,5]. The epithelium itself provides a layer of defense by protecting the deep tissue underneath the mucosa [1,6]. In the epithelial defense layer, tight junctions between specialized mucosal cells act as physical barriers to bacterial infection and noxious substance infiltration (Figure 1A). Rich blood supply and perfusion is important in maintaining mucosal homeostasis and protecting gut epithelium. In the submucosal layer, mucosal blood supply shuttles away noxious components that penetrate deep mucosal layers, as well as providing nutrients that maintain healthy mucosa through self-renewal [1]. The sub-epithelia level of defense is central for localization of wound macrophages, which play an essential role in gut mucosa homeostasis.

Homeostasis can be disrupted by weak intestinal integrity leading to inflammation and tissue damage [7]. There are multiple pathologic states that can lead to intestinal injury. Shearing forces such as surgical incisions, lacerations, and other acute wounds usually can heal without intervention. Bacterial infections with *H. pylori* are responsible for 90% of duodenal or gastric ulcers [1]. Often proton pump inhibitors (PPIs) are administered alongside anti-inflammatory drugs in ulceration cases to assist in healing. Long-term use of PPIs can lead to infections, impaired absorption of nutrients, dementia, kidney disease, accelerated bone loss, and possibly cancer [8,9]. Drugs such as non-steroid anti-inflammatory drugs (NSAIDs) can lead to mucosal injury through a wide array of mechanisms in both the upper and lower gastrointestinal mucosa [1]. NSAIDs can cause injury in the upper GI by cyclooxygenase (COX-1) inhibition. This inhibition results in a decrease in the secretion of prostaglandins, mucus, and bicarbonate, decreasing the hydrophobic buffer between the mucosa and the lumen of the alimentary tract [1]. Perfusion and blood flow through the epithelium usually increase in response to an irritating stimulus. NSAIDs inhibit prostaglandins, leading to less perfusion within the epithelium, which can worsen ischemic events, cause greater damage, and increase inflammation in the upper GI [1,6]. In the lower GI, NSAIDs can damage mucosa by binding bile acid, which increases its damaging effects on the mucosa and influences the gut microbiota. Its interaction with the microbiota changes the composition in favor of a Gram-negative heavy population, which activates Toll-like receptors (TLRs), increasing inflammation in the intestine [1,10].

The pathology of insufficient wound healing manifests in inflammatory bowel diseases (IBDs). IBD is a broad term that describes non-infectious, chronic, and inflammatory disruption of various areas throughout the digestive tract [11]. The etiology of IBD is a source of some confusion. It is an idiopathic disease where there is an abnormal immune response to either normal gut flora or epithelial tissue itself, an impaired epithelial mucosal barrier, and dysbiosis [12]. There are two common diseases that fall within the broader IBD term, both idiopathic. Ulcerative colitis is described by diffuse inflammation of the colonic and rectal mucosa. Crohn’s disease can be characterized by chronic, transmural, and segmental ulceration and inflammation of the digestive tract. Each disease can be classified by the severity and location within the digestive tract [12]. Some of the disease etiology may be attributed to chronic inflammation, which can develop when wound healing is compromised, ultimately leading to loss of epithelial barrier function [13]. IBD treatment therapies include, but are not limited to, 5-aminosalicyte, antibiotics, corticosteroids, and immunosuppressants [1,14]. A greater emphasis on mucosal healing has emerged as a goal in clinical management of IBDs in recent years [14]. Greater understanding of mucosal homeostasis and its healing mechanisms will give us a better grasp on pharmacologically targeting these cascades which could assist in the healing process and allow for better prognosis.

Quick and efficient healing of the epithelial barrier upon injury is essential. Mucosal healing is mediated by different cell types that are within the mucosa itself. First platelets are recruited to the injury site where clotting occurs (Figure 1B). Epithelium wound closure is coordinated by proliferation and epithelial sheet migrations that help close injured tissue [15]. For small injuries, usually about eight cells wide, the purse string method of healing is utilized. Actin cables form parallel to the wound’s edge. Contraction that produces tension on these actin cables leads to the closure of the wound, bringing adjacent cells together allowing for the formation of cell–cell connections [1,16].

This form of wound healing is much like suture closure of a circular wound, with suture material acting as the actin cables. Wounds that are larger than this, however, require a mechanism known as epithelial restitution. Depending on the depth and size of the wound, this process takes time to heal, requiring multiple overlapping processes of migration, proliferation, and angiogenesis [1,17]. The development or redifferentiation of the intestinal epithelium to a migratory phenotype is necessary for the closure of large wounds. This redifferentiation is the result of intracellular cascades that ultimately lead to the activation of an epithelial migratory phenotype and is dependent on proteins such as FAK and Rac1. FAK and Rac1 mediate the disassembly of cell–cell connections at sheet edges, as well as regulate actin dynamics during sheet migration [15]. After migration, redifferentiation must occur back into specialized enterocytes and colonocytes associated with the intestinal epithelium and its absorptive biological function.

Cytokines and immune-induced environments are also important in the process of intestinal wound healing (Figure 1C). Epithelial restitution is known to be mediated by IL-10 and IL-22. IL-22 has been shown to have regenerative and innate defense abilities. IL-22 is secreted by activated T cells, NK cells, and CD11^+^ leukocyte cells [18]. IL-22 treatment within mouse models increases the expansion and proliferative properties of Paneth cells within villi crypts [19]. IL-22 has also been shown to increase mucous production in goblet cells, which ultimately improves and protects intestinal membrane function [19]. In the following paragraphs, we discuss in detail how IL-10 induces a favorable anti-inflammatory microenvironment by mediating macrophage polarization events based on external stressors. This ever-changing environment may be a key to the pathology of IBDs.

## 2. FAK Activation and Its Role in Intestinal Healing

**Figure 2:**
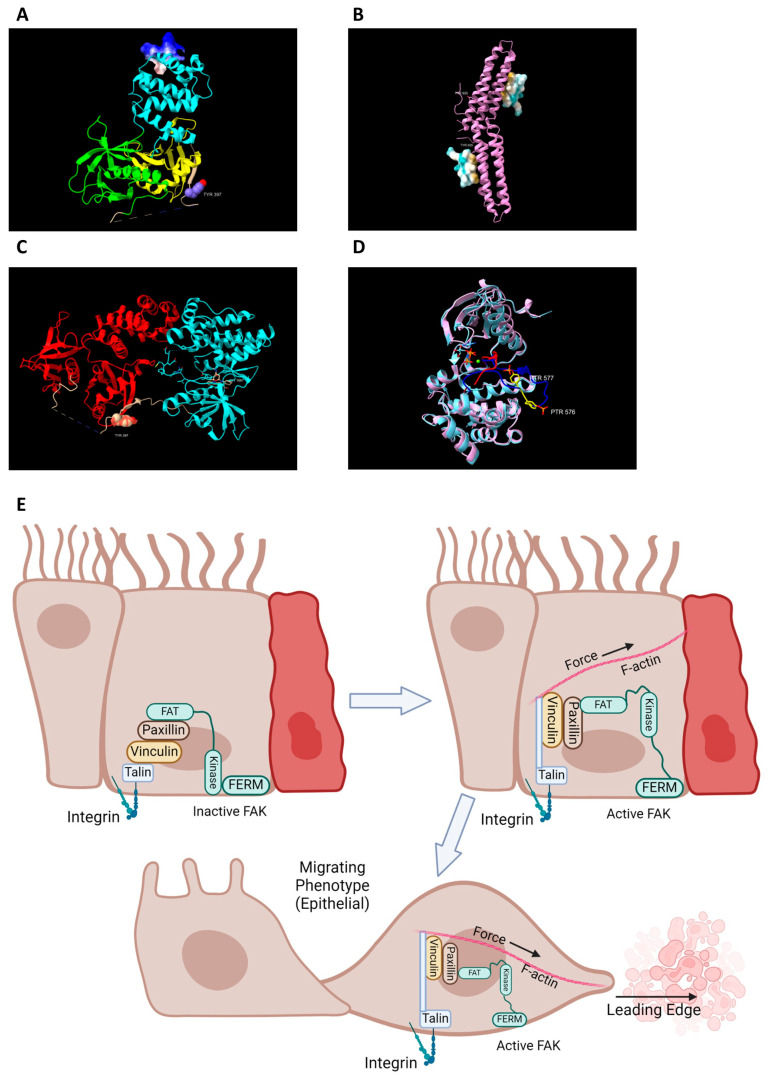
Multiple domain regions of FAK (**A**) Crystallographic structure showing FAKs’ FERM domain. Shown in yellow is the F1 lobe (residues 33–127), the F2 lobe (residues 128–253) is colored cyan, and the F3 lobe (residues 254–361) is colored green. Shown in purple is the FAK activating phosphorylation sight Tyr397 within the tan colored linker region. The basic patch KAKTLRK (residues 216–222) within the F2 lobe is shown as an electrostatic surface. This patch has been shown to be extremely important for membrane binding to phosphatidylinositol 4,5-biphosphate (PIP2) along the inner leaflet of FAs. (**B**) Structure of the FAK FAT domain with the Paxillin LD4 domain bound on two interaction sights of the domain. The top FAT structure shows Tyr925, a phosphorylation sight of Src within helix one, in stick structure. Paxillin is bound via hydrophobic interactions (shown as yellow surface) to helices 2 and 3. The bottom FAT domain shows a second binding sight for paxillin along helices 1 and 4. The same Src phosphorylation sight Tyr925 is labeled and is shown to be interacting with paxillin at a binding pocket. (**C**) Structure shows autoinhibited FAK with association and interaction between the kinase (Cyan) and the FERM (Red) domains. Shown in stick form is the activation loop of the kinase domain. Bound to the active site is staurosporine analog AFN941. This rendering has Tyr397 in the linker region with red surfaces showing that the hydroxyl group of the tyrosine is sequestered from solution, making its phosphorylation difficult in the inhibited conformation. Tyr397 is also shown to be requisitioned away from the kinase active site, by around 35 angstroms. (**D**) Rending comparing FAKs active kinase (Pink) and FAKs inactive kinase (light blue). FAKs activation is largely contributed to a conformational change in the activation loop after DFG motif, which is seen in the superimposition. The relative structure remains the same, however, with a root mean square deviation (RMSD) of 1.44 angstroms. Shown within the activated kinase activation loop (blue) are phosphorylated tyrosines 576 and 577 (yellow). Inactive kinase loop (red) does not show tyrosines as their structure was not elucidated. AMP-PNP, an ATP analog, is shown bound to the active sight along with a catalytic Mg^2+^. (Chimera X [32,33,34] was used by the first author to generate all protein structures.) (**E**) After injury, FAK is recruited to the cellular membranes of boarder intestinal cells. Integrin plays a key role in cell–extracellular space communication and providing friction for cellular movement. Talin is recruited to open conformation integrins and acts as a structural protein along with vinculin and paxillin to mediate FAK interaction as well as actin polymerization to allow for the development of a leading edge. The force produced by treadmilling actin along the leading edge is what induces a migratory phenotype and epithelial sheet migration. Created with BioRender.com (accessed on 20 June 2024).

FAK is a multiple domain protein. The N terminal region contains a band 4.1-ezerin-radixin-moesin (FERM) domain [1] (Figure 2A). This domain is essential for localizing FAK to the plasma membrane and is required due to its function as a linker protein between the plasma membrane and the cell cytoskeleton [20]. This interaction is needed in allowing the cell to generate force at its focal adhesion (FA) points inducing cell movement. The focal adhesion targeting (FAT) domain acts as a scaffolding protein, which allows the protein to localize and stabilize within the cell due to its interactions with paxillin [1,21] (Figure 2B). These two domains work together as a framework and are essential in cell movement and force generation; these domains also allow the protein to localize within the cell, producing a variety of responses [1]. The central kinase domain triggers a variety of downstream signals once activated, including the Ras/Raf/MAPK [1,22,23,24,25,26], P130Cas-Crk [1,27,28,29,30], and Phosphatidylinositol 3-kinase (PI3K)-AKT pathways [1,31]. FAK also contains three linker domains with proline-rich regions (PRs) which are essential in binding Src homolog (SH) regions on other proteins [1]. These structural and catalytic features of FAK allow the protein to carry out its important role in establishing FAs and mediating cell movement.

FAK has multiple phosphorylation sites, which allow for activation of the protein and establishing maximal catalytic activity. Tyrosine 397 (Y397) is a key autophosphorylation site that activates FAK [1]. FAK displays itself in an autoinhibited form that shields Y397 from phosphorylation. Binding to surface receptors or other soluble proteins or molecules is necessary for FAK to unfold and expose Y397 for autophosphorylation [1] (Figure 2C). These protein–protein interactions allow FAK to become autophosphorylated at Y397 by reconfirmation of the protein [1,35].

Phosphorylation of Y397 attracts Src binding via an SH2 binding domain and to a PXXP sequence within the PR1 region of FAK via an SH3 binding domain [1,36]. Src phosphorylates Y576 and Y577 within the FAK catalytic domain, which allows FAK to achieve full catalytic capacity [1] (Figure 2D). Another phosphorylation event occurs at the FAT domain on Y925, and this leads to the disassembly of the FAK from focal adhesions (FAs), terminating FAK’s activation [1]. The Y925 phosphorylation allows Grb2 binding and initiates the disassembly of FAK from FAs. There are more phosphorylation sites that are less known but may have profound importance for FAK activity that will need to be explored in the future.

Epithelial sheet migration plays a crucial role in intestinal healing and homeostasis. Acquisition of an epithelial migratory phenotype is essential in epithelial sheet migration, and it is mediated by FAK activity due to its role in assembling and disassembling FAs. Activated FAK becomes associated with FAs within the cell’s membrane via protein interactions between the FAK FAT domain and paxillin [1,35] (Figure 2B).

The localization of FAK to the membrane allows the assembly of FA points, which generate force via cytoskeletal contraction [37]. Fast-moving cells are characterized by small “point-like” FAs that are not considered to be mature [37]. Mature FA complexes play more of a role in adhesion than in migration, these FAs present themselves as a larger streak like protein aggregates along the cell’s membrane [37].

The activation and transition into a motile phenotype are regulated by FAK and the Rho family of GTPases. FAK induces signaling cascades that are ultimately regulated by the Rho family, which is a part of the Ras GTPase super family [38]. Activation of the Rho family cascades triggers transcription factor activation of c-myc, c-Jun, c-Fos, and/or ETS, which transcribe proteins that have a role in proliferation, survival, angiogenesis, and differentiation [39,40,41], and lead to the reorganization of the cell’s cytoskeleton, inducing epithelial sheet migration [37,38,42]. Once an epithelial migratory phenotype is achieved, however, the amount of total FAK and the amount of active FAK decreases [37]. This nuance makes FAK an extremely desirable target for pharmaceutical intervention when faced with a case of resistant ulcer or intestinal injury. Much is still yet to be known about FAK and the various roles that it plays within the cell.

## 3. Small FAK Activating Drugs

Targeting FAK Y397 for phosphorylation via small pharmaceutical molecules could have medical implications for chronic mucosal injuries [37,43]. ZINC40099027 is a small, water-insoluble molecule that increases FAK phosphorylation and activation [44]. Another small molecule, M64HCl, which is water-soluble, was found to have the same effect in Caco-2 cells [45]. M64HCl is enterically absorbed and has drug-like properties, making it an attractive candidate as a pharmacological agent in treating chronic and acute intestinal injury. The exact mechanism of how these drugs increase the activation state of FAK is unknown. The complex and partially unknown structure of FAK makes it difficult to propose an activation mechanism.

Along with FAK activation, these two drugs increased wound closure, which was observed using monolayer wound closure assays. Wound closure was significantly greater after treatment with M64HCl in comparison to vehicle control [45]. These novel drugs have a multitude of implications other than treating acute intestinal mucosal injuries. As stated before, IBD is a collection of diseases that disrupts the homeostasis of epithelial healing due to chronic inflammation pathology [46]. Drugs such as M64HCl could potentially shift the equilibrium of epithelium healing, giving patients with IBD a better prognosis.

## 4. Role of FAK in Chemotaxis of Immune Cells

Murine FAK knockout immune cells, particularly macrophages, have reduced motility and migration, indicating that FAK plays a key role in immune cell chemotaxis, which is necessary in intestinal immunity and healing [47]. Invading pathogenic species in intestinal injuries express factors such as fibronectin, CpGs, proteoglycan (PGS), lipopolysaccharide (LPS), and PolyI:Cs. These factors induce pathways through Toll-like receptors (TLRs), which control cell mobility, by controlling pathways involving FAK activation and SRC binding [47,48]. Quick movement of immune cells towards damaged sites is integral to intestinal healing.

Integrins play a key role in intracellular signaling within macrophages through their binding potential to ECM scaffolding proteins [47]. Proteins such as fibronectin, collagen, and laminin regulate cells via intracellular signaling cascades, which can lead to a wide variety of changes within the cell [48]. The activation of TLRs stimulates an activation cascade that is dependent on the TLR and its bound ligand [48]. Some TLRs induce effects from the plasma membrane such as TLR4 and TLR2, whereas others such as TLR3 and TLR9 are endocytized and signal from an intracellular endosome. These cascades activate NF-κB, increasing the expression of inducible nitric oxide synthase (iNOS) [48]. Through an unknown mechanism, iNOS secondary messengers (NO) lead to the association of FAK and SRC [48]. This association leads to increased cell mobility, but conversely the activation of FAK was observed at a phosphorylation event on tyrosine 861 (Y861) instead of Y397 [48]. While FAK has been a well-known protein that is necessary for cell migration in epithelial cell lines, it is now an integral component of immune cell migration as well [47,49,50].

Digiacomo et al. identified a signaling cascade that utilizes tyrosine kinase colony stimulating factor-1 receptor (CSF-1R) involving FAK as a messenger [47]. Under steady state conditions, CSF-1 (M-CSF) acts as its substrate and initiates motion, proliferation, and survival within macrophages through its binding to CSF-1R. When fibronectin binds to plasma membrane integrins, there is an induction of a FAK-dependent signaling cascade that is similar to CSF-1 binding to CSF-1R [47]. Fibronectin binding was shown to initiate FAK-mediated phosphorylation of Y723 on CSF-1R, which increased mobility in macrophages [47]. Digiacomo et al. then proposed a mechanism where fibronectin and integrins interact extracellularly to then permit FAK activation at Y397 [47]. This allows for SRC family kinase binding and maximal FAK catalytic activity to be achieved [47]. Downstream GTPases, Rac1 and Cdc42, then contribute to motility and cell polarization events within the cell [47].

## 5. Macrophage Polarization and Response in Intestinal Healing

Innate immunity and macrophage polarization play a crucial, yet not completely understood, role in mucosal healing and homeostasis. Macrophages are highly plastic cells with a wide spectrum of endotypes. General monocytes (Mφ) polarize into either classical M1 (Ly-6C^high^ CCR2^high^ CX3CR1^low)^ or non-classical M2 (Ly-6C^low^ CCR2^low^ CX3CR1^high^) phenotypic cells. These cells are polarized by environmental factors, foreign antigens, and immune mediating proteins, which aids in the establishment of intestinal mucosa homeostasis and healing [51].

M1 polarized cells produce a pro-inflammatory microenvironment [51]. M1 cells are derived from peripheral monocytes that polarize in accordance with cytokine and growth factor concentrations that are present within the tissue of the infiltrating monocyte [52,53,54]. Increasing concentrations of molecules such as LPS and TNFα act as signaling molecules that bind Mφ receptors and initiate polarization into M1 [55]. Cellular signaling cascades activate transcription factor NF-κB, which is a master regulator of M1 gene expression [52,56].

The LPS-induced M1 polarization phenotype occurs through binding and activating Toll-like receptor 4 (TLR4). Then phosphoinositide 3-kinase (PI3K) is activated, which induces phosphorylation events on the lipid membrane, and recruitment and activation of AKT1/2 [52]. AKT activation inhibits tuberous sclerosis complex (TSC) via phosphorylation [57]. This inactivation of TSC leads to activation of the transcription factor mTOR1 [52]. AKT1 and AKT2, however, are shown to have differing intracellular signaling cascades. AKT2 leads to the activation of NF-κB and induced expression of M1 genes, while AKT1 leads to an inhibition of M1 gene expression [52].

Recruitment of M1 polarized cells is essential in tissue damage responses. Molecules such as DNA, RNA, or ATP from lysed cells act as signaling molecules and attract immune cells to the site of damage through chemotaxis [58]. Due to the M1 cells’ ability to recognize phosphatidylserine markers on apoptotic cells, the removal of damaged tissue and then initiation of proper healing can occur [52,59]. The increased populations of M1 cells during healing can explain some pathophysiologies if they are not properly regulated by M2 polarized cells.

M2 cells produce an opposite, anti-inflammatory microenvironment that is associated with a repair phenotype [52,60,61]. Signaling cytokines IL-4 and IL-13 favor polarization of a M2 phenotype through a STAT6-dependent manner [60,61]. M2 cells overexpress protein markers, arginase 1 (Arg-1), and the mannose receptor (CD206). They have an increased secretion of chemokines CCL17 and CCL22, as well as IL-10, which help to establish an anti-inflammatory environment within the gastrointestinal tract [61]. IL-10 aids in intestinal healing through its suppression of pro-inflammatory responses from M1 macrophages [62]. IL-10 also plays a role in mucosal healing at the enterocyte level by triggering proliferative pathways [63]. IL-10 binds to its receptor IL-10Rα, which leads to a phosphorylation event on transcription factor cAMP element binding proteins (CREB) at serine 133 via Protein Kinase A (PKA) [64] in a STAT3-dependent manner [63]. The activation of CREB allows for the expression of WNT-1-induced signaling protein 1 (WISP-1) to increase within epithelial cells [63]. WISP-1 is then secreted and acts as a downstream effector that leads to activation of proliferative cascades, aiding in mucosal healing by promoting the upregulation of genes such as POU5F1 (OCT4) and NANOG [63].

At the site of injury, nitric oxide (NO) is produced largely by immune and nonimmune cells and promotes inflammation in surrounding tissue due to its toxic effects [65]. M2 cells decrease NO production by shunting metabolites, especially L-arginine, away from NO production by inducible nitric oxide synthase (iNOS) and towards urea and ornithine production by Arg-1 [52]. This shuttling of metabolites by enzyme expression is a key factor in decreasing the inflammatory effects that macrophages exhibit [52]. Overall, a greater ratio of M2 to M1 cells reduces the inflammation that is accompanied with mucosal injury and creates a more nurturing environment for mucosal healing [51].

## 6. Waterfall Effect: Continuum of Macrophage Differentiation in Intestinal Healing

Epithelial damage leads to an increased flux of circulatory monocytes entering the mucosal tissue initiating the healing response [66]. During a normal injury, monocytes infiltrating the intestinal tissue will differentiate into macrophages and polarize into M1 cells [58,67]. This allows for the clearance of debris from the wound site and a mounted immune response to the influx of bacteria from the gut lumen into the mucosal tissue. This first phase of intestinal healing is important, but if it is prolonged, healing of the injury is more difficult and will lead to a chronic wound.

The recruitment and timing trends of the differing macrophage phenotypes has long been an enigma; however, there has been an effort to determine the infiltrating macrophage origins and differentiation in response to epithelial injuries. Wound-associated macrophages (WAMs) are not resident macrophages, but instead are recruited to injured areas from circulating monocytes [68]. Determining the morphology of phenotypically different monocytes and macrophages requires characteristic markers that change with phenotype. Ly-6C and CCR2 are macrophage surface markers that are characteristic of undecided monocytes usually found in circulation [68,69,70]. Ly-6C is present in nearly 50% of bone marrow-derived immune cells [69]. A high concentration of this receptor makes cells more apt to differentiate into the monocyte lineage. There is a fluctuation of the Ly-6C concentration throughout monocyte and macrophage morphologic changes [69]. The high concentrations of this receptor are present in M1 polarized macrophages, while a lower concentration is observed in M2 polarized cells [67]. CCR2, another characteristic marker of morphologic change, is essential for adherence of immune cells to the vasculature of the intended tissue [70]. Much like Ly-6C, CCR2 is seen in higher concentrations in undifferentiated monocytes and M1 macrophages, but in lower concentrations in M2 polarized macrophages [67]. These differences may be attributed to CCR2’s function in macrophage recruitment to intended sites.

CCR2 is a G-protein coupled receptor (GPCR) that mediates chemotactic responses and is essential in the recruitment of WAMs to sites of injury [70]. It is a member of the CCL2-CCR2 axis, which proves to be a critical interaction for the recruitment of monocytes from the blood stream into surrounding tissue [67]. The monocyte recruitment from circulation is essential for proper healing [58,67]. CCL2 or monocyte chemoattractant protein-1 (MCP-1) is expressed and secreted in the endothelium, epithelium, and bone marrow [71]. This small protein acts as a potent chemoattractant for monocytes, T lymphocytes, and natural killer (NK) cells [71]. The activation of CCR2 subsequently leads to the activation of adenylyl cyclase via the receptors alpha (α) subunit [71]. The beta and gamma (βγ) subunits promote nuclear localization of NF-κB to the nucleus via AKT activation [71]. These subunits also lead to RAS/RAC activation, prompting the activation of p38, c-Jun N-terminal kinase (JNK), and extracellular signal regulated kinase (ERK) [71]. Activation of these pathways leads to increased expression of c-myc, c-Jun, c-fos, and CREB genes [71]. AKT and p38 activities also allow for the activation of integrins on monocyte membranes, which is essential in rolling, adhesion, and migration through the endothelium [71,72].

There is evidence of a waterfall effect that occurs in the lamina propria, accounting for the diversification of intestinal macrophages. The waterfall effect refers to a continuum of changes from monocyte to macrophage [67]. This continuum of macrophage differentiation may be key to understanding the pathology of inflammatory bowel diseases (IBDs) and other inflammatory diseases. Prolonged exposure to aggressive phenotypes could lead to chronic inflammation within the intestine. For example, monocytes maintaining a pro-inflammatory phenotype instead of cascading through gradual changes and adopting an anti-inflammatory phenotype can cause unnecessary damage to surrounding tissue, creating various undesirable symptoms. Flow cytometry has shown that CCR2^high^ monocytes and CCR2^low^ monocytes were seen adhering to the vasculature, which could be indicative that both monocyte phenotypes—M1 and M2—come from the blood stream [67]. It was also shown using CX3CR1^GFP^ CCR2^RFP^ mice that there were subtle increases in CX3CR1 and decreases in CCR2, adding evidence to the waterfall continuum and gradual change in phenotype over time [67]. Both pieces of evidence suggest two different mechanisms. The first is that the continuum is due to the recruitment of specific circulatory monocytes that have predetermined differentiation upon recruitment. The second is a phenotypic change to previously recruited macrophages, which accounts for the change that is seen throughout the continuum as time progresses. Many papers lean towards the second hypothesis having a larger impact on macrophage population continuum [58,66,67]. This means that while the macrophage population will renew itself, over time infiltrating macrophages will become resident macrophages that play a key role in maintaining the mucosa homeostatic environment.

## 7. Resident Macrophages Function in Maintaining Epithelial Integrity

Once obtained from circulation, resident macrophages themselves are found mainly in the intestinal lamina propria and within the Peyer’s Patches of the ileum. These resident cells are positioned right below the epithelial layer and are involved in epithelial housekeeping, monitoring for pathogenic bacteria, and the clearance of senescent cells [66,67]. Supporting evidence by De Schepper et al. demonstrated that a “firewall” of macrophages from a resident lineage added an additional layer of protection between the gut lumen and underlying tissues from bacterial infection [73]. This wall of macrophages regulates what can access the portal blood stream from the gut lumen [67]. What is evident is that there is crosstalk between this macrophage layer, microbiota, the mucosal epithelial cell layer, and the endothelial layer, helping coordinate symbiosis between the intestinal components. This cooperation is essential in maintaining an intestinal barrier with high fidelity for essential nutrients and reduced permeability to noxious agents and pathogens.

Proper cycling of macrophage phenotypes also plays a large role in maintaining the high integrity of the epithelial barrier. It has been shown that chronic exposure to inflammatory cytokines disrupts the epithelial barrier, making it more permeable to unwanted or harmful agents [74]. Kim et al. explored mechanisms by which cytokine milieus can be manipulated to maintain the tight junction barrier and its low permeability state. Inflammatory induction with LPS to co-cultured colon epithelial Caco-2 cells and RAW macrophages followed by treatment with cinnamon extract led to higher transepithelial electrical resistance, decreased tight junction permeability, and a lower concentration of pro-inflammatory cytokines [75]. The growing knowledge on the plasticity and activity of macrophages, along with the microenvironments that they induce, is a promising area of research for future discoveries in mucosal healing and overall health of the mucosal barrier.

## 8. GM-CSF as a Factor for Intestinal Healing

Granulocyte macrophage colony stimulating factor (GM-CSF) plays an important role in hematopoiesis, as well as proliferation and differentiation of granulocytes and macrophages [76,77]. It has been known since the 1970–80s that the colony stimulating factor (CSF) family comprises important cytokines in mounting immune responses upon exposure to pathogens. The father of modern hematology, Don Metcalf, discovered that macrophages and granulocytes could be grown in agar, forming colonies [78]. This discovery launched a quest to identify and purify the cytokine that was responsible for the formation of these colonies [78]. A decade of hard work came to fruition as Don Metcalf and his team were able to sequence and purify first GM-CSF and then all the other CSFs [78]. Today, GM-CSF is a ubiquitous molecule that is secreted by many tissue types. A variety of non-hematopoietic tissues also contain the receptor complexes capable of binding GM-CSF. The cellular signaling and responses are still not completely understood in many organ systems, including the brain, lungs, and intestinal tract [79,80,81].

The GM-CSF receptor is a two-subunit receptor that is made up of an alpha subunit (GM-CSFRα, CD116), which acts as the binding domain, and a beta domain (GM-CSFRβc, CD131), which acts as the signaling domain [79,82,83]. The signaling beta domain is shared with the alpha cytokine specific domain receptors for cytokines IL-3 and IL-5 in humans [79,82]. Key downstream signals from the GM-CSFR involve JAK2/STAT5 and ERK, with ERK being linked to human monocyte survival in vivo [82]. GM-CSF cytokine binds the alpha domain with low affinity, forming a binary complex [83]. A high-affinity complex is formed once the beta domain binds the binary complex that is formed between GM-CSF and the alpha domain; this forms a hexamer complex [83]. The binding of the cytokine to each receptor is dependent on specific interactions at four different sites, including interfaces between both receptor domains and the cytokine itself [83]. Receptor interactions are also required, as a dodecamer complex needs to be formed by the hexametric complexes, forming a dimer, for signaling to occur [83]. Once active conformation is achieved, the beta subunit acts as a binding site for JAK2, triggering the JAK/STAT pathway through interaction between the JAK2 FERM domain and the beta domain cytoplasmic tail [84]. Dimerization of activated receptors allows for transphosphorylation and pathway induction [83].

In response to antigen presentation, dendritic cells (DCs) and macrophages produce IL-23 and IL-1β to mount an inflammatory response [85,86]. In response to these secreted cytokines, ILC3s secrete IL-22, which binds to intestinal epithelial cells (IECs), improving the epithelial layer’s integrity as well as producing antimicrobial peptides (AMPs) and GM-CSF [85,86]. As a target for immune cells, GM-CSF controls the activation of several key cytokines, including tumor necrosis factor (TNF) and IL-1β, through augmenting macrophage polarization and activation, alongside its primary role of proliferation and survival [85]. It was also shown that genes regulated within macrophages by GM-CSF signaling included inflammasome activation (ll1β, Hifla, Nlrp3), prostaglandin E2 production (Ptgs2), and co-stimulation (CD40, CD80) [85]. This gives us a glimpse into the mechanisms of how GM-CSF coaxes macrophages towards certain microenvironment generation and initiates immune responses. This is, however, an oversimplification, and, like many physiological findings, requires more explorations towards understanding that there is a dose- and time-dependent effect of GM-CSF that persuades monocyte lineages [82].

Low concentrations of GM-CSF lead to phosphorylation of S585 on the beta domain of the GM-CSFR on immune cells, which results in cell survival regulation via phosphoinositide-3-kinase activity [82]. Small doses of GM-CSF (0.3 ng/mL) cause downstream pathways to disable inflammatory functions and acquire suppressor cell characteristics [82,87]. These suppressor cell characteristics only occurred on monocytes that were recruited to inflammatory sites. T cell secretion of GM-CSF and IFNγ led to activation of the monocytes. Continued exposure of GM-CSF at concentrations higher than 0.3 ng/mL led to abrogation of the activated response and initiated suppressor monocyte activity [87] The licensing of monocyte-derived suppressor cells is dependent on the AKT/mTOR/mTORC1 pathway activation followed by signaling through the IFNγR/IRF-1 pathway [82,87]. Higher concentrations lead to activation and polarization events, alongside increasing secretion of chemokines and cytokines. All latter processes are indicative of a mounting immune response [82]. These known monocyte/macrophage activation differences in response to GM-CSF concentration pose an interesting area in which to pursue further exploration within the realm of inflammatory bowel diseases, and the role that macrophages play in gut mucosa homeostasis.

Even though epithelial cells secrete GM-CSF, another contributor to the GM-CSF concentration gradient that is responsible for macrophage polarization is Group 3 Innate lymphoid cells (ILC3s) [85]. Upon dextran sodium sulfate (DSS)-induced colitis, ILC3s were the only cell type to show dynamic change in GM-CSF production, supporting its role in regulating macrophage and DC responses [85]. GM-CSF helps regulate macrophages and DCs by initiating retinoic acid release [88]. Increased concentration of retinoic acid regulates T cells and their role in maintaining homeostasis and increasing tolerance towards certain microbiota [85,88].

The GM-CSF-mediated crosstalk between innate and adaptive immune cells creates a positive feedback loop, which regulates the relationship of intestinal epithelial homeostasis and the bacterial species that occupy the lumen of our intestines. While GM-CSF is critical for crosstalk between gut microbiota and the body’s immune system, it also produces an inflammatory response [85]. ILC-depleted mice showed less of an inflammation response and disease severity based on murine colon weight and colon shortening several days after treatment with DSS [85]. These mice did, as expected, exhibit a greater bacterial burden compared to wild-type mice [85]. These findings demonstrate that GM-CSF may be an important contributor to the pathologies of diseases such as IBD and a M1 polarizing factor [55]. The inflammatory environment prompted by this polarization event are imperative in immune responses but could surely lead to pathologies if prolonged. The total effect of GM-CSF is difficult to determine due to its wide variety of targeted tissues. Even though it has been determined that GM-CSF receptors are expressed on intestinal epithelial cells [82], the further downstream signaling pathways that respond to secreted cytokines are largely unknown, along with the effects that they induce.

There is some evidence that shows that GM-CSF plays a role in intestinal healing by impacting the intestinal epithelium directly. GM-CSF^−/−^ mice compared to wild-type (WT) mice showed resistance to proliferation and healing when treated with DSS [79]. Treatment with 3% DSS impacted the colon of WT mice, but they were eventually able to recover from the ulceration induced by the DSS [79]. For GM-CSF^−/−^ mice, 3% DSS was lethal, and these mice showed extensive weight loss, epithelial baring, and fecal blood loss [79]. When exogenous GM-CSF was administered to GM-CSF^−/−^ mice, there were improvements in ulceration and bleeding, showing that the delay in healing was due to low endogenous GM-CSF [79]. The GM-CSF was produced and secreted by an unexpected source. Using bone marrow chimeras, the GM-CSF source responsible for the increase in mucosal healing was not produced by myeloid derived cells, but instead GM-CSF was produced by the epithelium itself [79]. This source of GM-CSF production proposes an autocrine signaling pathway that leads to increased proliferation and healing [79]. Mice lacking the signaling domain for the GM-CSF receptor also showed increased ulceration and bleeding upon DSS treatment [79]. This autocrine signaling event is widely unknown and may be a key in understanding mucosal healing, as well as mucosal integrity, permeability, and inflammatory events.

The complex role that GM-CSF plays in its regulation of immune cell activation and epithelial healing makes it an excellent target for further research. The differences in tissue targets, signaling pathways, and responses pose a lot of questions about GM-CSF activity in the gut. There is more that needs to be uncovered in the role GM-CSF plays in aiding epithelial and immune system homeostasis.

## 9. Conclusions

**Figure 3:**
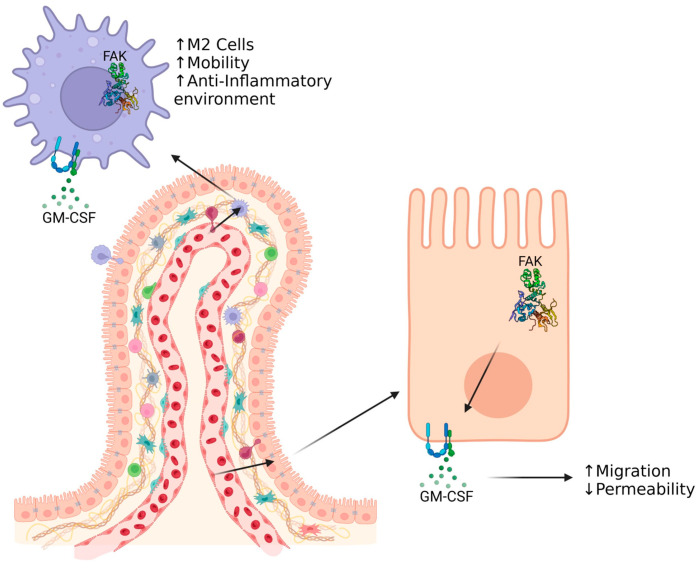
Schematic of FAK and GM-CSF involvement in intestinal epithelial cells and macrophages during intestinal wound healing. The activation of FAK and the binding of GM-CSF to the GM-CSF receptor initiates pathways in the epithelial intestinal cells that promote increased epithelial sheet migration and decrease intestinal permeability. Likewise, in macrophages, FAK activation and GM-CSF binding increase macrophage mobility, M2 macrophage polarization, and an anti-inflammatory environment. Created with BioRender.com (accessed on 27 June 2024).

Intestinal homeostasis is dependent on the activation and protein activity of FAK (Figure 3). A quick turnover of FAs is associated with a mobile phenotype and allows epithelial sheet migration to occur after injury. Maturation of FAs is correlated with an adhesion phenotype, which may play a role in protecting gut mucosa against injury. The structure of FAK is complex and contains many globular domains, phosphorylation sites, and linker regions, which contribute greatly to the protein’s function. Its enzymatic activity also plays a large role in generating intracellular signaling cascades. The use of small drug-like activator molecules such as ZINC40099027 or M64HCl may soon allow for pharmaceutical intervention that targets wound healing in epithelial cells [37,43,44,45,89].

FAK has also proven to be an important protein in macrophage/monocyte chemotaxis, as well as having a role in intracellular signaling (Figure 3). Macrophages/monocytes have an important role in the regulation of inflammation upon injury. They may also be an integral component in the pathology of IBDs. Approximately 2.39 million Americans struggle from a variety of IBDs that affect most of the alimentary tract from the stomach to the colon [90]. With nearly 1 in 100 Americans affected, the main causes of IBDs are still largely unknown [90]. It has been shown that infiltration of macrophages into gut mucosa play a critical role in immunity due to the important chemokines that they secrete. CSFs and macrophage polarization are fundamental in maintaining gut homeostasis and aiding in intestinal wound healing, but their dysregulation may also be an underlying cause for inflammatory issues that occur after injury and in IBDs.

Of these important signaling molecules, colony stimulating factors have been shown to be important not only in stimulating the intestinal immune system, but they may also play an important role in assisting in mucosal healing. The crosstalk between the epithelium and the immune system presents an intricate and largely unknown field of study. New discoveries are being made every day that fill in these unknowns and add to a growing knowledge that will lead to a greater understanding of our biology.
